# Supplementary material for: Conformation-Dependent Influences of Hydrophobic Amino Acids in Two In-Register Parallel β-Sheet Amyloids, an α-Synuclein Amyloid and a Local Structural Model of PrPSc
Source: ACS Omega. 2022 Aug 24;7(35):31271–88. doi: 10.1021/acsomega.2c03523 (PMC9453792; doi:10.1021/acsomega.2c03523)
Supplement: Supplementary file 1 — ao2c03523_si_001.pdf [file ao2c03523_si_001.pdf]

Supporting Information

Conformation-dependent influences of hydrophobic amino acids in two in-register parallel β-sheet amyloids, an α-synuclein amyloid and a local structural model of PrP^{Sc}

Hiroki Otaki,[*,†,‡] Yuzuru Taguchi,[¶,‡,§] and Noriyuki Nishida[¶]

†*Center for Bioinformatics and Molecular Medicine, Graduate School of Biomedical Sciences, Nagasaki University, 1-14 Bunkyo-machi, Nagasaki 852-8521, Japan*

‡*Contributed equally to this work*

¶*Department of Molecular Microbiology and Immunology, Graduate School of Biomedical Sciences, Nagasaki University, 1-12-4 Sakamoto, Nagasaki 852-8523, Japan*

§ *Present address: Kakunodate General Hospital, Senboku-shi, Akita 014-0394, Japan*

E-mail: otaki@nagasaki-u.ac.jp

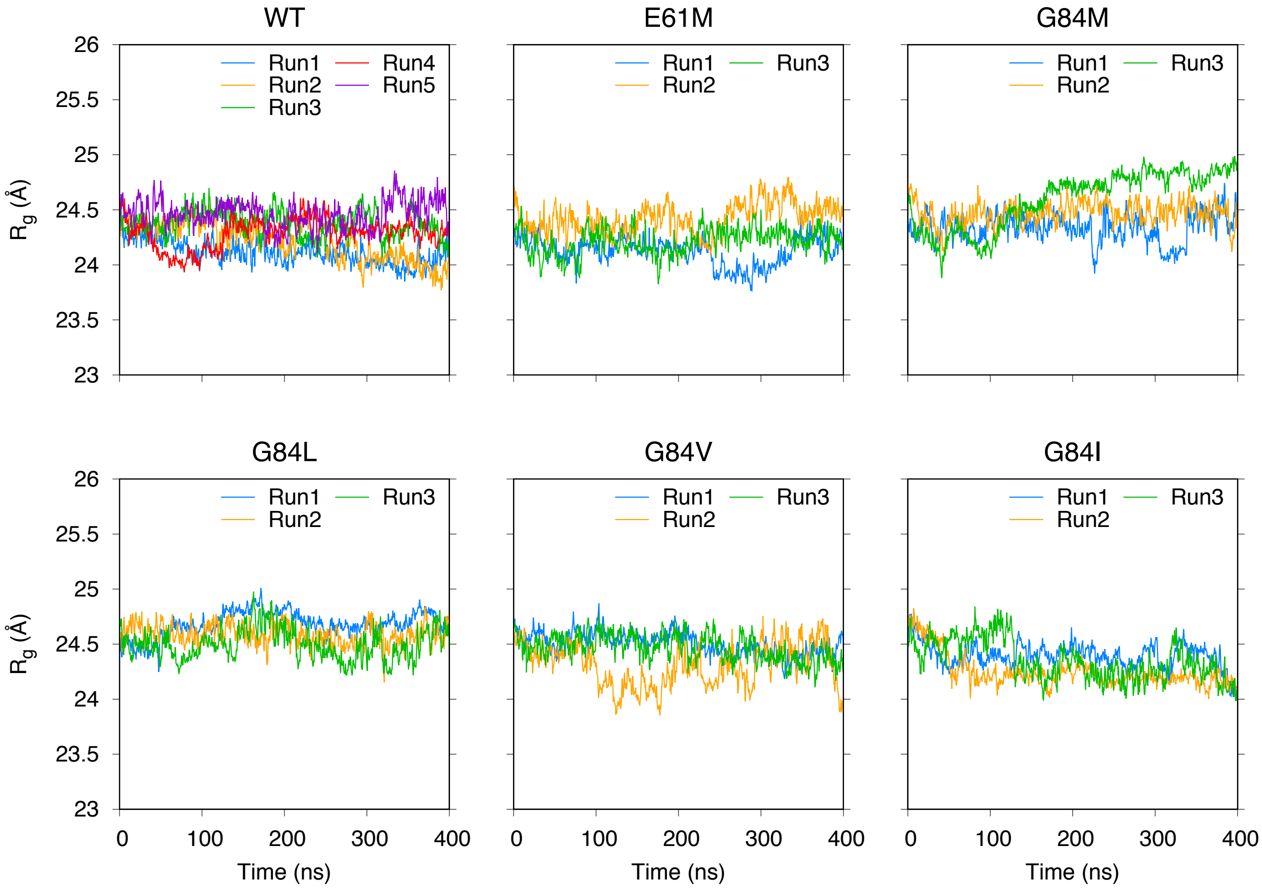

Figure S1: Radius of gyration (R_g) as a function of time for the αSyn mutants.

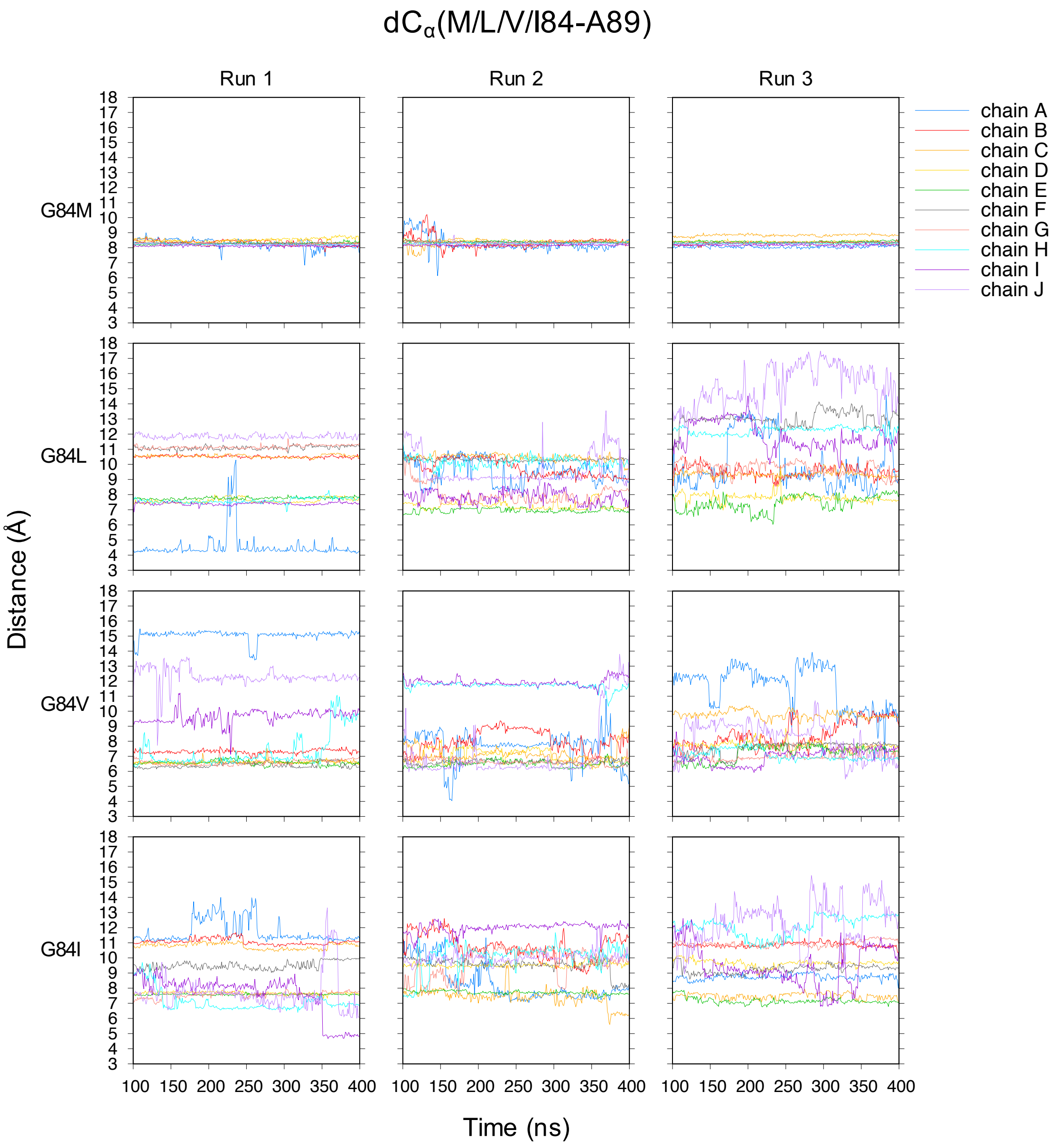

Figure S2: Time series of distance between C_α atoms (dC_α) of residues M/L/V/I84 and A89 in the αSyn mutants.

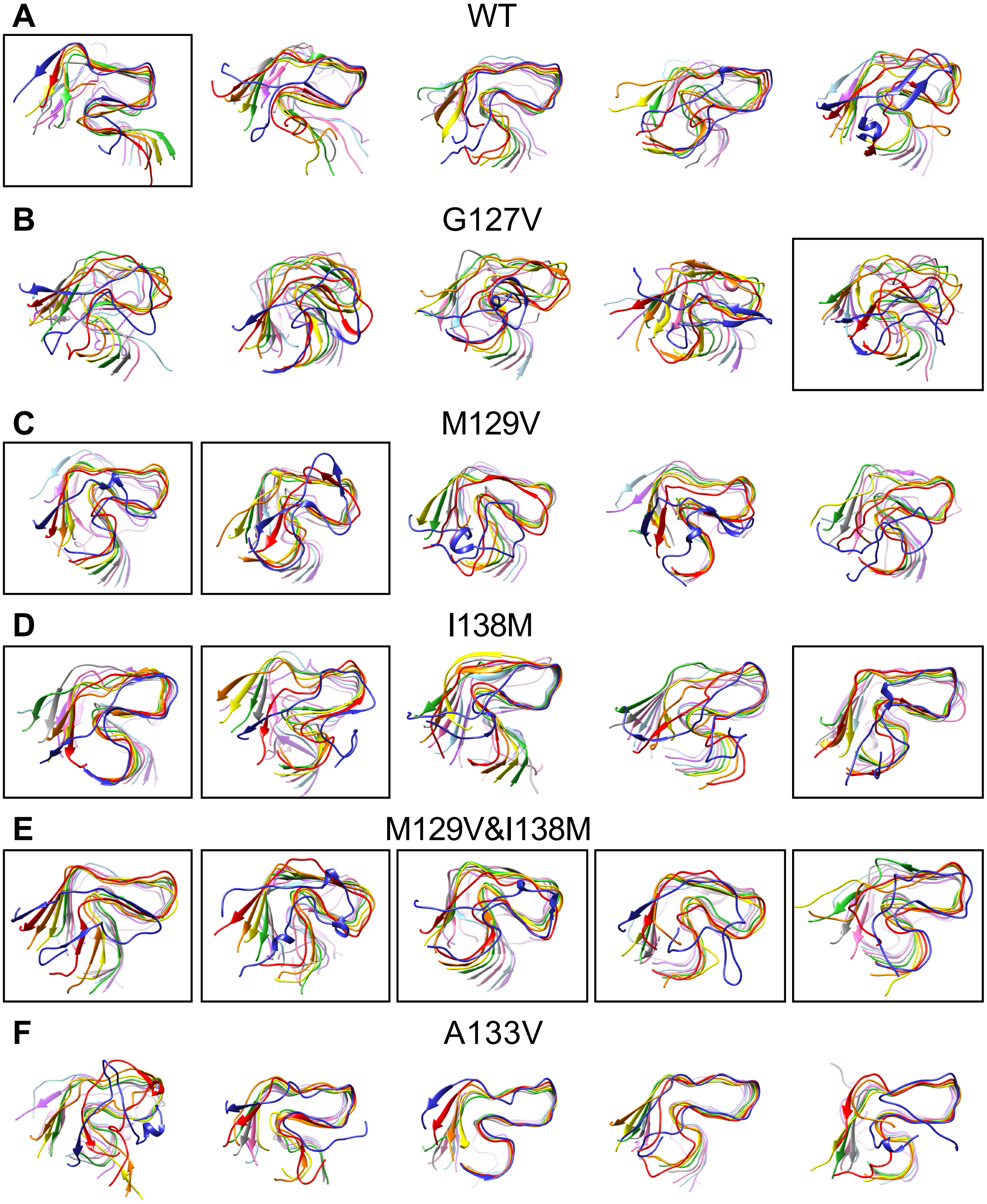

Figure S3: Final snapshots of the five independent runs of MD simulation of $PrP_{107\text{-}143}$. (A) WT, (B) G127V, (C) M129V, (D) I138M, (E) M129V&I138M, and (F) A133V. From left to right, top to bottom: Runs 1–5. Those with proximity scores for A133-P137 higher than 200 are in black boxes. See also Figure 10 in the main text.

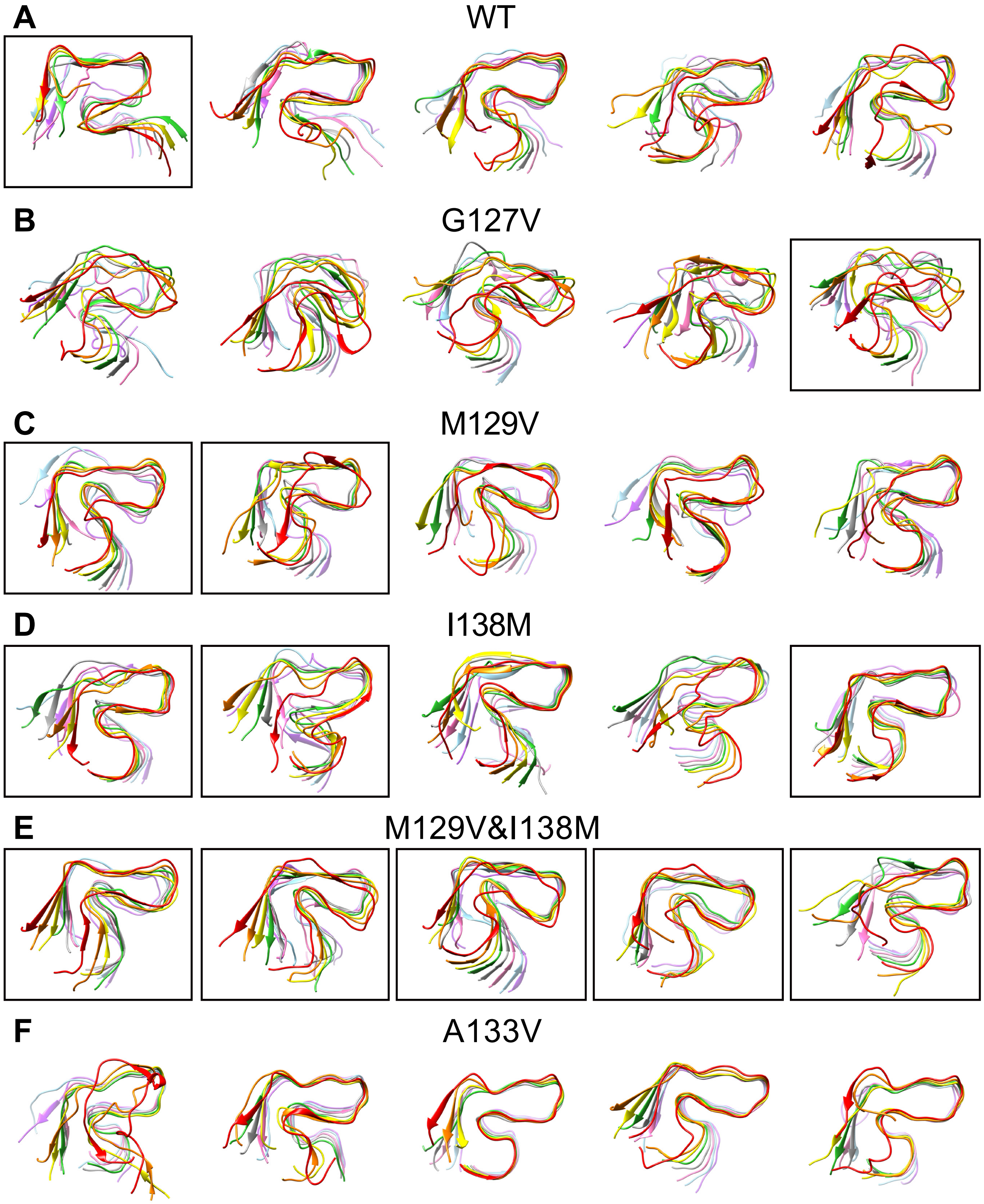

Figure S4: Identical to Figure S3, except that chains A and J are removed for clarity.

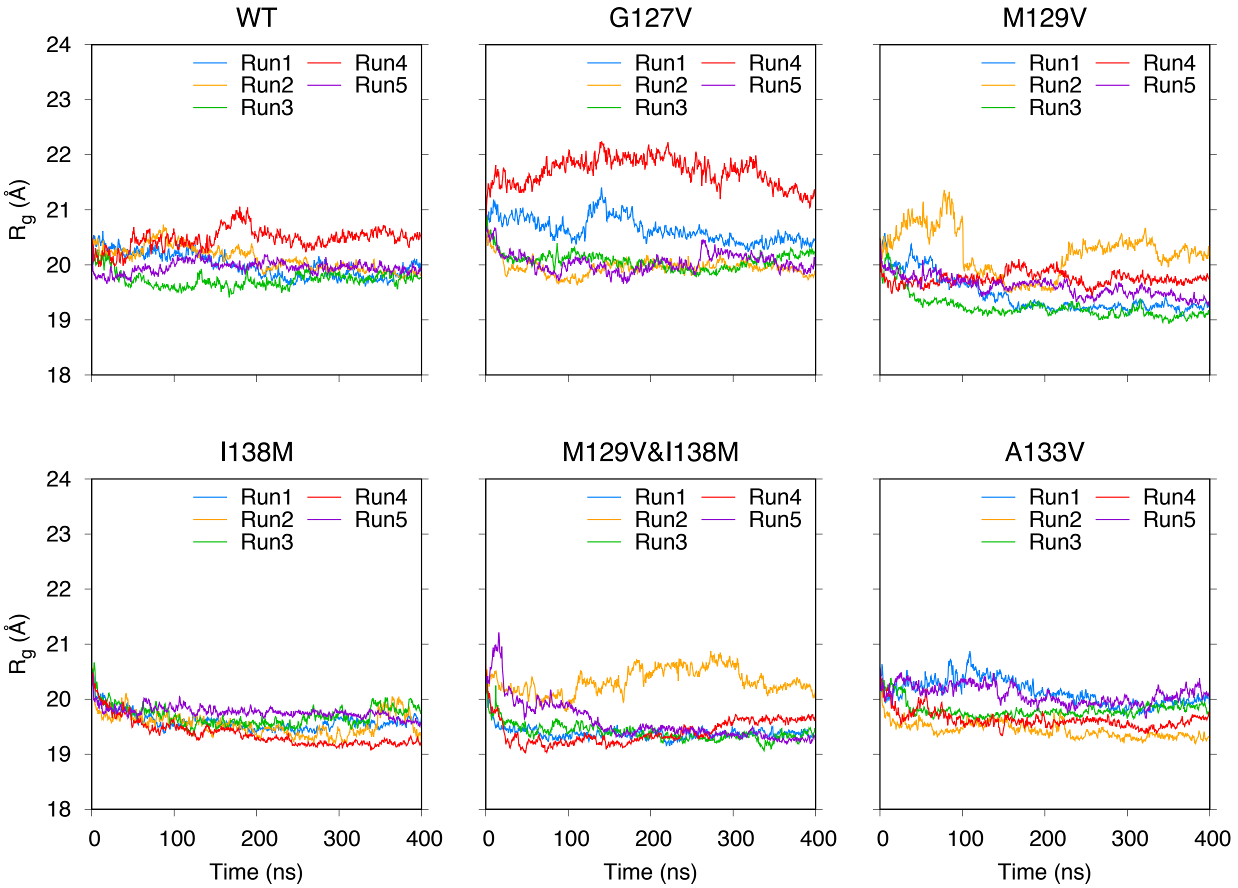

Figure S5: Identical to Figure S1, except for the $PrP_{107\text{-}143}$.

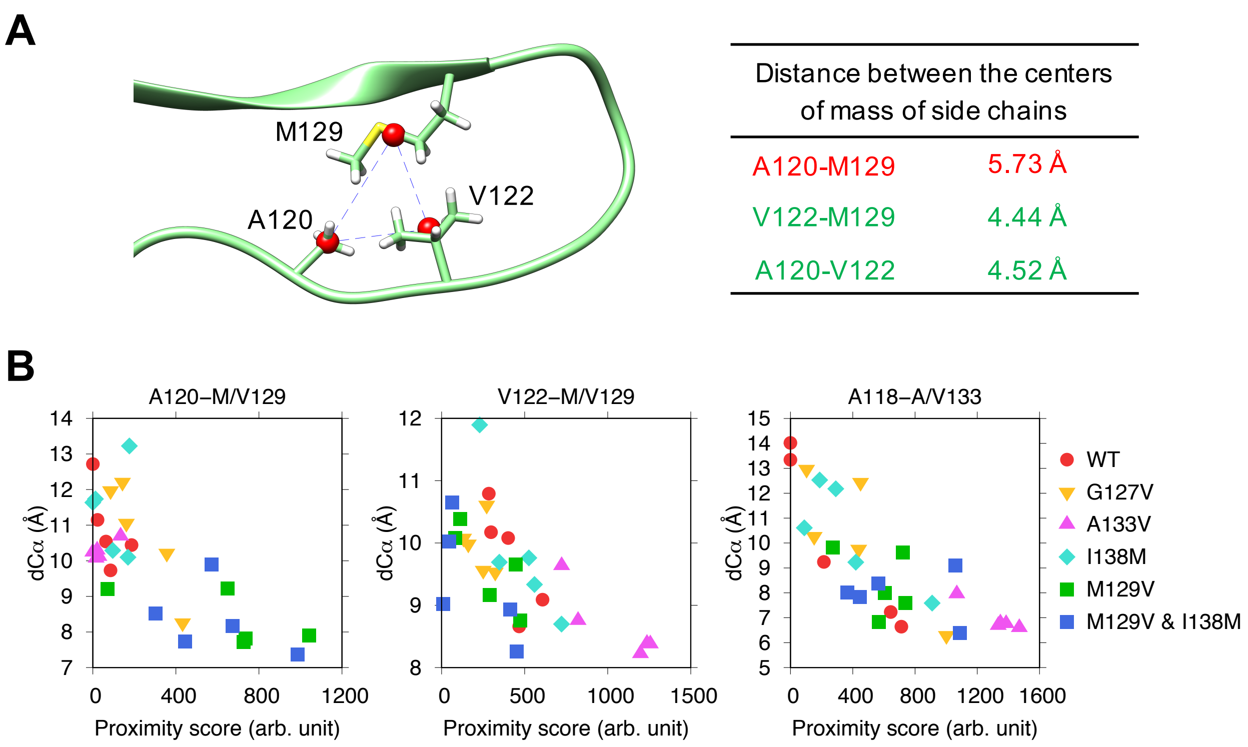

Figure S6: (A) Definition of hydrophobic contact and proximity score. A "hydrophobic contact" is counted when the distance between the centers of mass of side chains (red spheres in this figure) of two hydrophobic residues are within 5 Å.[1] For example, in this figure, V122-M129 and A120-V122 are counted as contacts but A120-M129 is not. A "proximity score" basically sums up the proportions of time/snapshots in a trajectory when hydrophobic contact is present between the two residues of interest over chains B–I. When a single residue has hydrophobic contact simultaneously with a residue on the same chain and another residue of the same residue number on the next chain, the score for the interaction in the snapshot is two. Therefore, the total proximity score can be larger than 800 if there are many inter-chain interactions. (B) Correlations between proximity score and dC_α between the corresponding residues averaged over eight chains in the last snapshot of MD simulation.

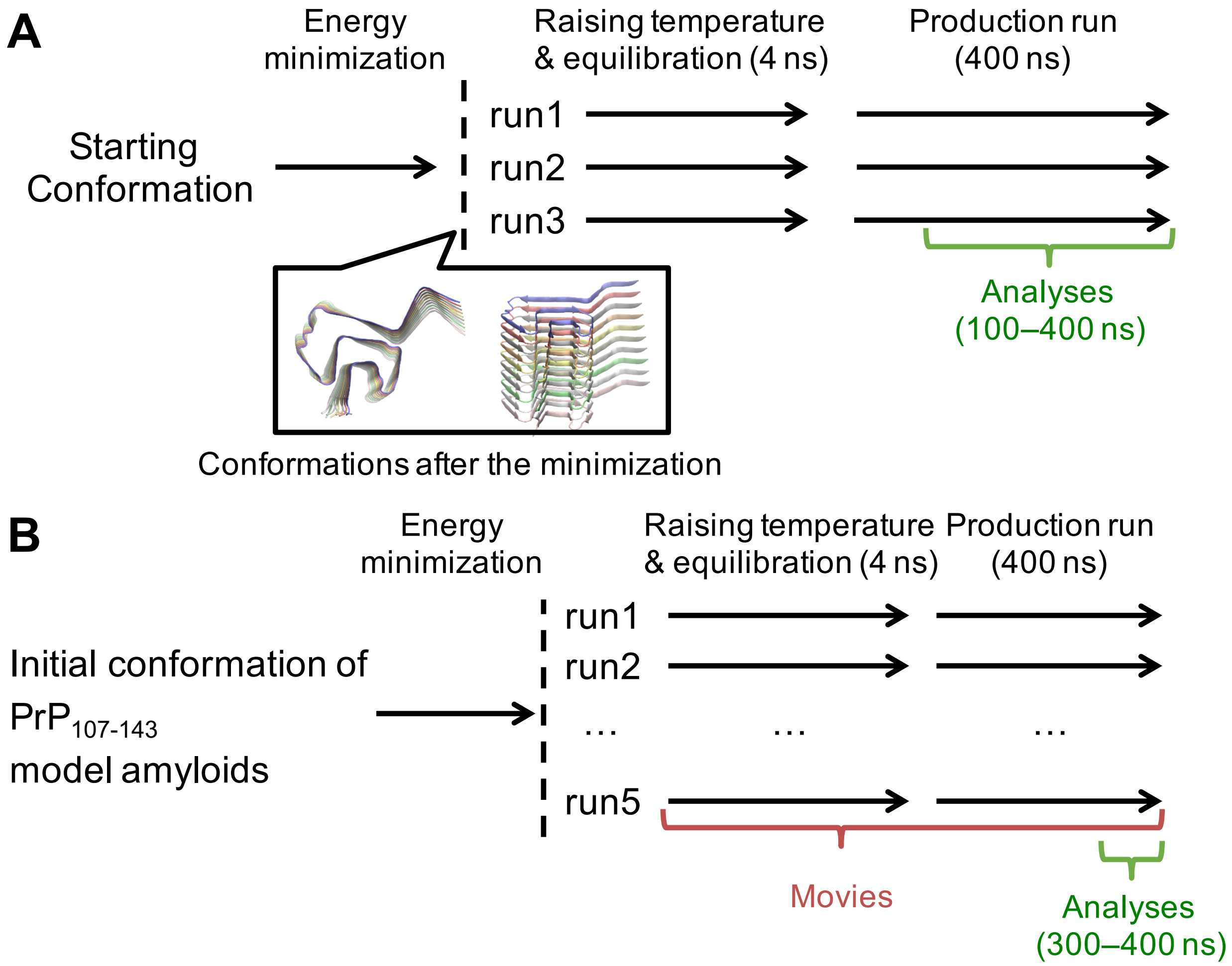

Figure S7: Procedure of MD simulation. (A) MD simulation of αSyn. (B) MD simulation of the tentative local structural model of PrP^{Sc}, $PrP_{107\text{-}143}$ amyloids.

Table S1: Root-mean-square inner product (RMSIP) and cumulative proportion of variance of αSyn calculated with the first 20 principal components

Run	WT		E61M		G84M	
	RMSIP	Cumulative %	RMSIP	Cumulative %	RMSIP	Cumulative %
1	0.74	69.4	0.72	85.4	0.74	83.3
2	0.70	74.9	0.69	75.8	0.75	77.7
3	0.73	74.8	0.79	74.4	0.76	80.8
4	0.72	74.0				
5	0.77	81.7				

Run	G84L		G84V		G84I	
	RMSIP	Cumulative %	RMSIP	Cumulative %	RMSIP	Cumulative %
1	0.75	69.7	0.73	74.0	0.69	81.7
2	0.68	81.1	0.69	81.1	0.65	75.6
3	0.64	74.7	0.73	72.0	0.71	81.0

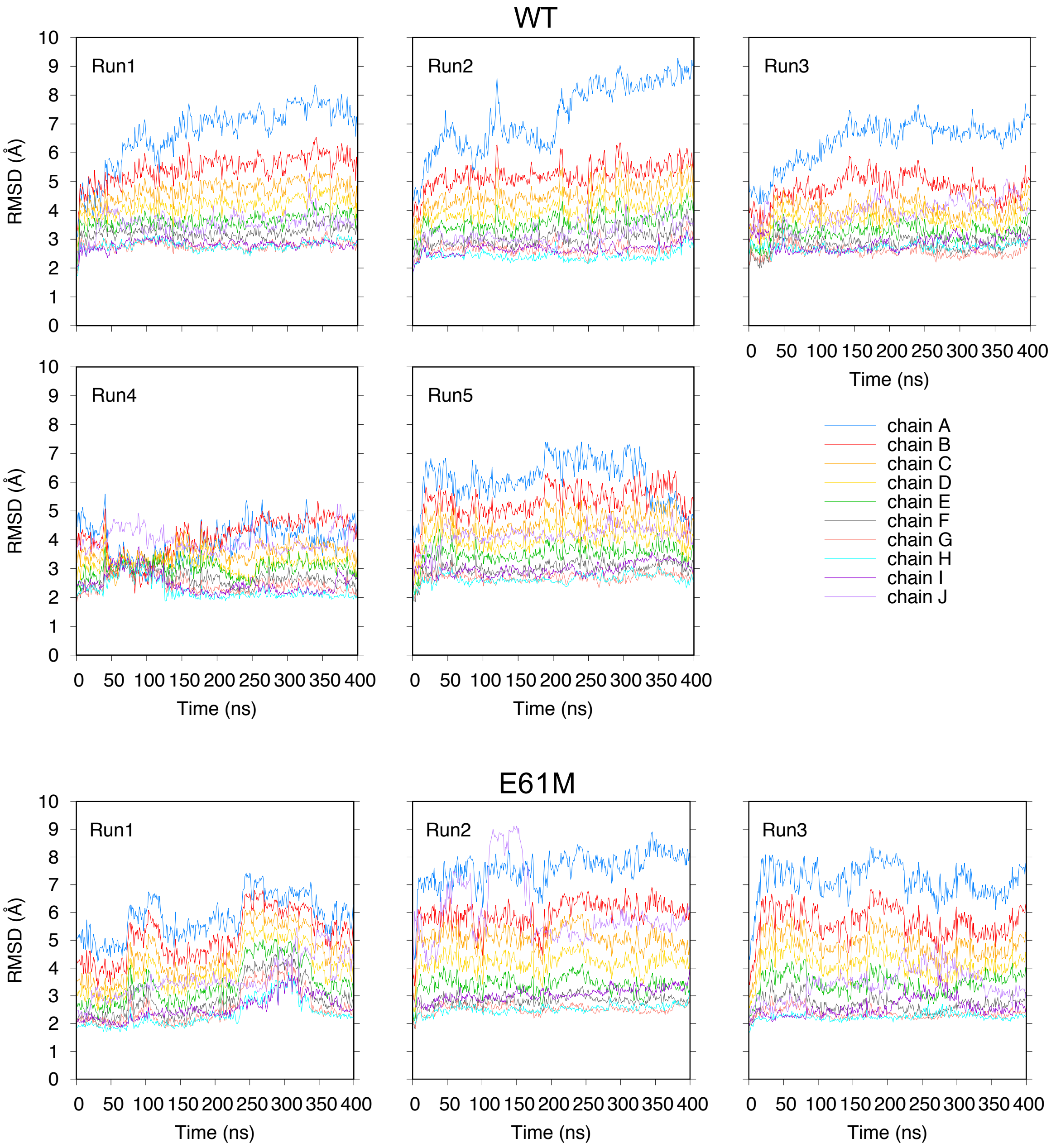

Figure S8: Backbone root-mean-square deviation (RMSD) of the αSyn amyloids with respect to the solid-state NMR structure (PDB ID: 2N0A[2]).

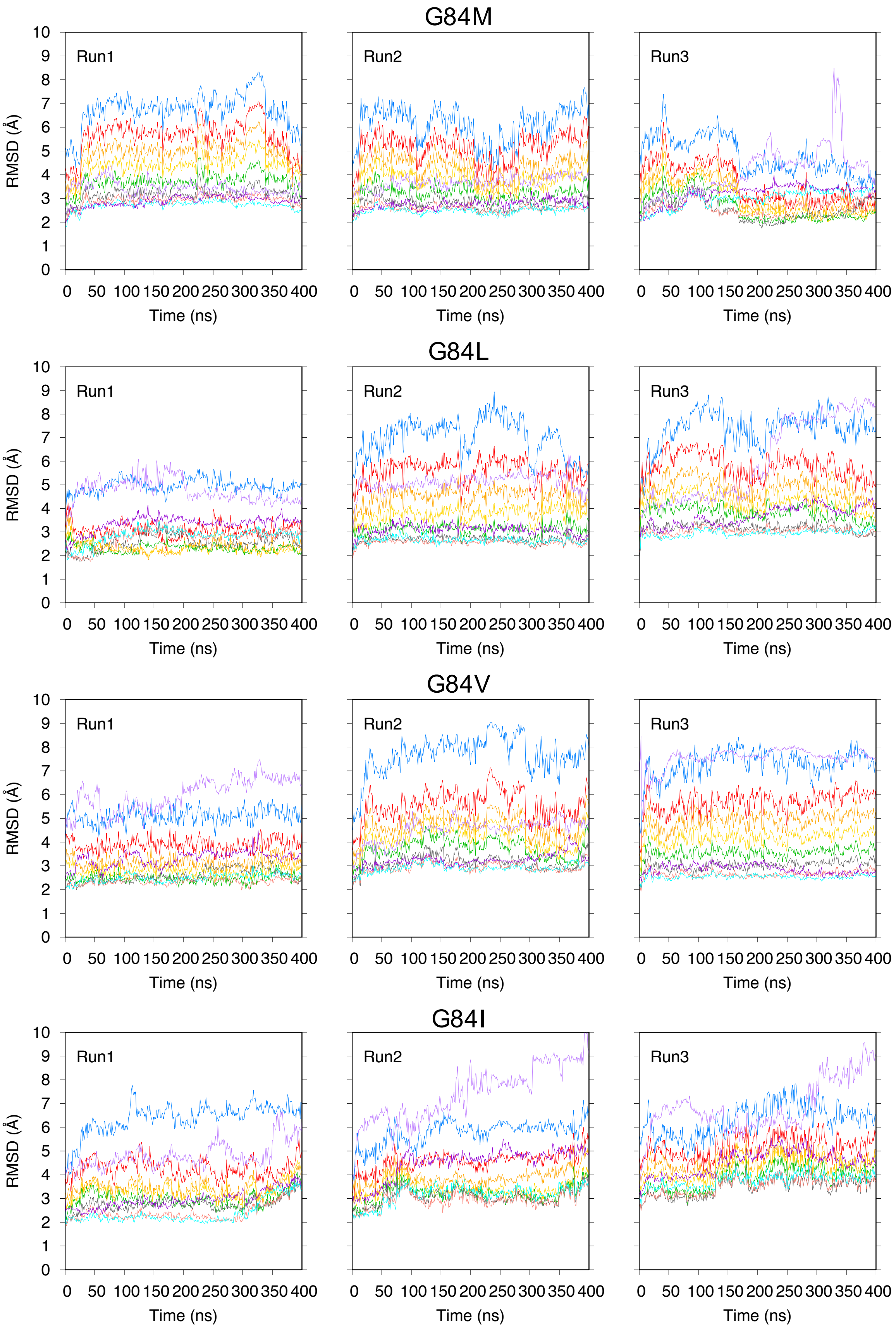

Figure S8: (cont'd.)

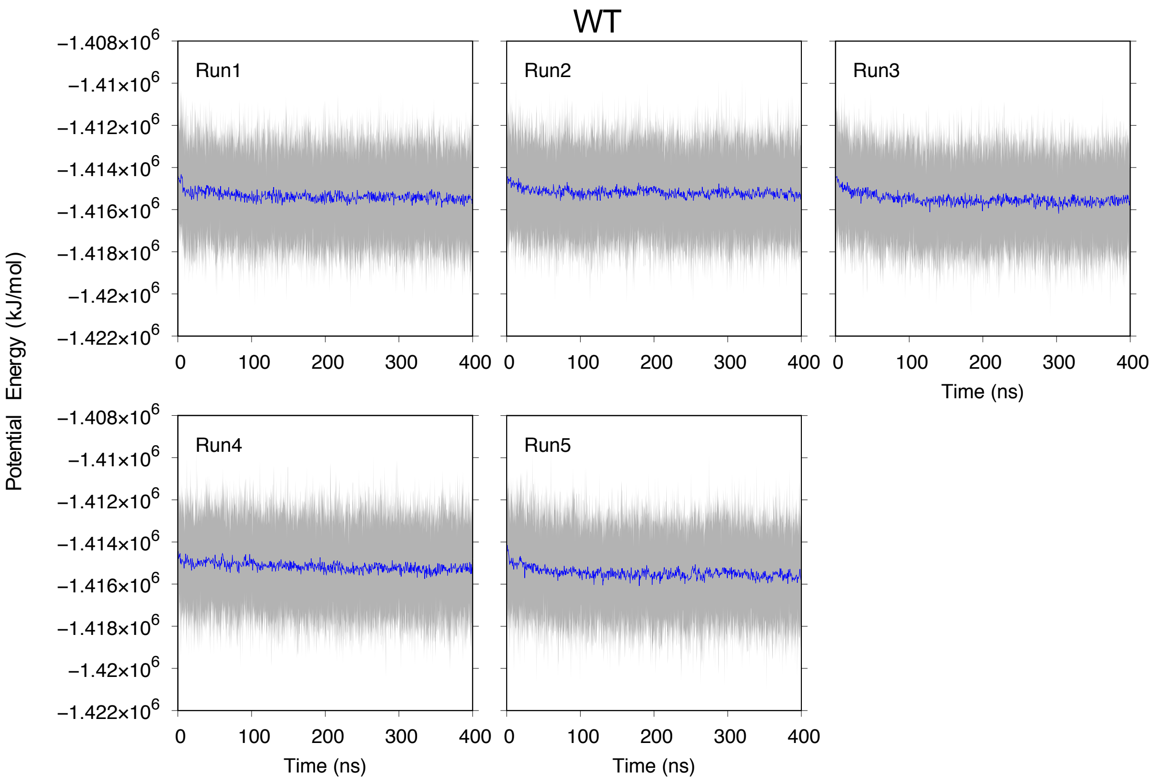

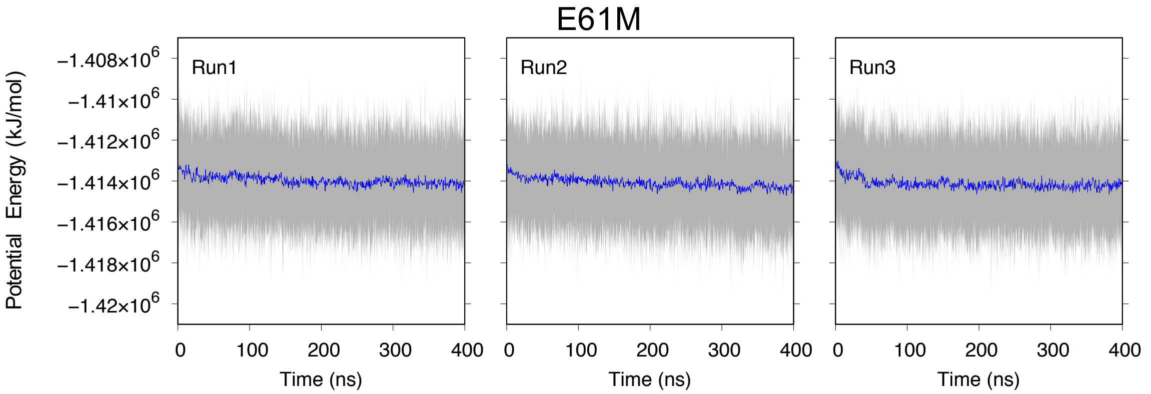

Figure S9: Potential energy as a function of time for the αSyn amyloids (gray). The blue line represents a running average over 500 ps.

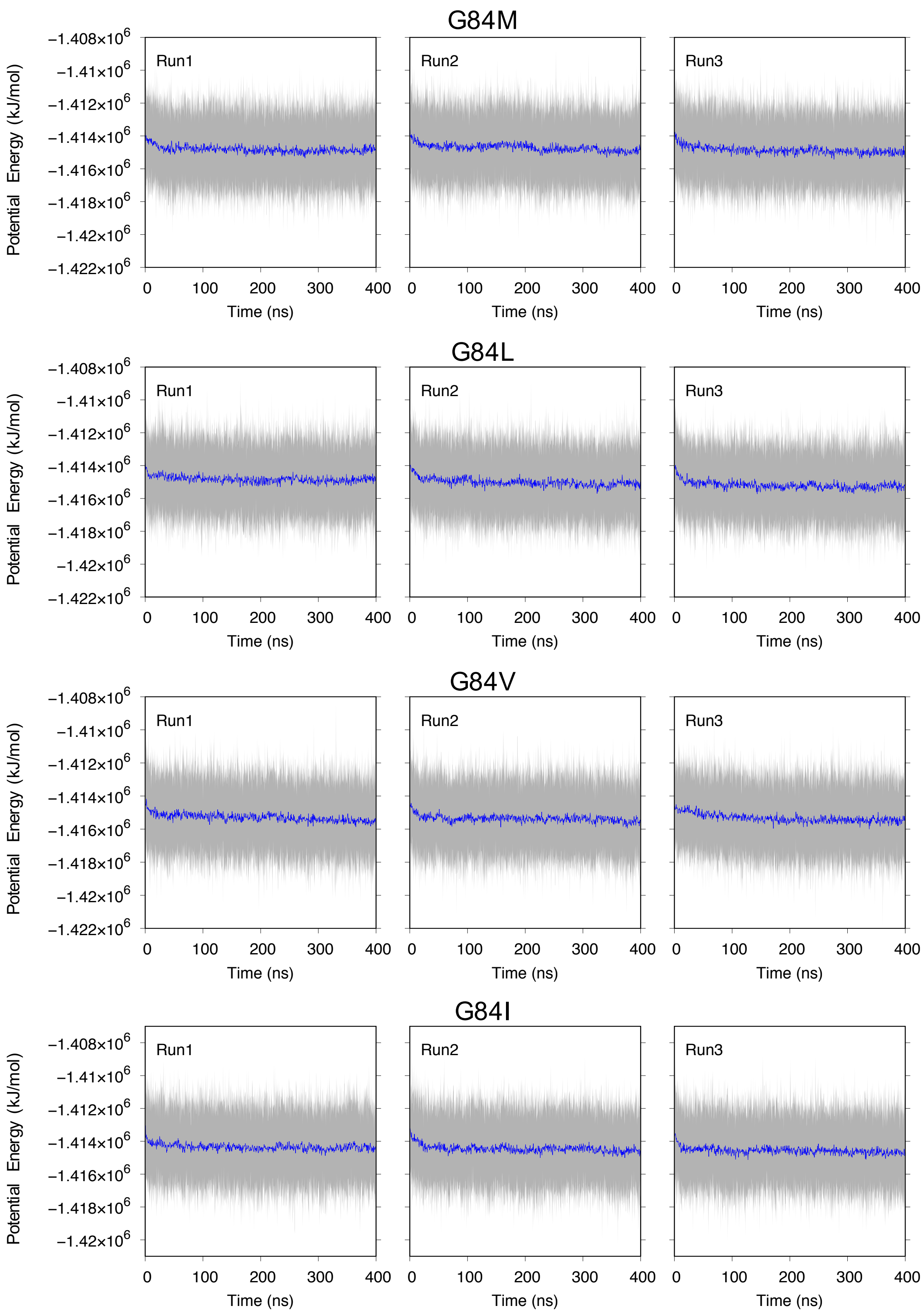

Figure S9: (cont'd.)

Table S2: Identical to Table S1, except for $PrP_{107\text{-}143}$

Run	WT		G127V		M129V	
	RMSIP	Cumulative %	RMSIP	Cumulative %	RMSIP	Cumulative %
1	0.69	79.9	0.67	71.3	0.69	75.0
2	0.61	75.8	0.70	66.3	0.65	75.0
3	0.70	79.8	0.65	73.5	0.65	67.6
4	0.68	75.2	0.66	78.0	0.71	74.1
5	0.71	77.2	0.68	68.2	0.65	73.8

Run	I138M		M129V&I138M		A133V	
	RMSIP	Cumulative %	RMSIP	Cumulative %	RMSIP	Cumulative %
1	0.70	75.4	0.64	66.5	0.67	76.9
2	0.70	85.6	0.66	74.7	0.68	75.8
3	0.68	79.8	0.66	79.0	0.75	70.7
4	0.70	69.8	0.62	75.6	0.72	76.3
5	0.66	71.2	0.73	63.1	0.64	84.0

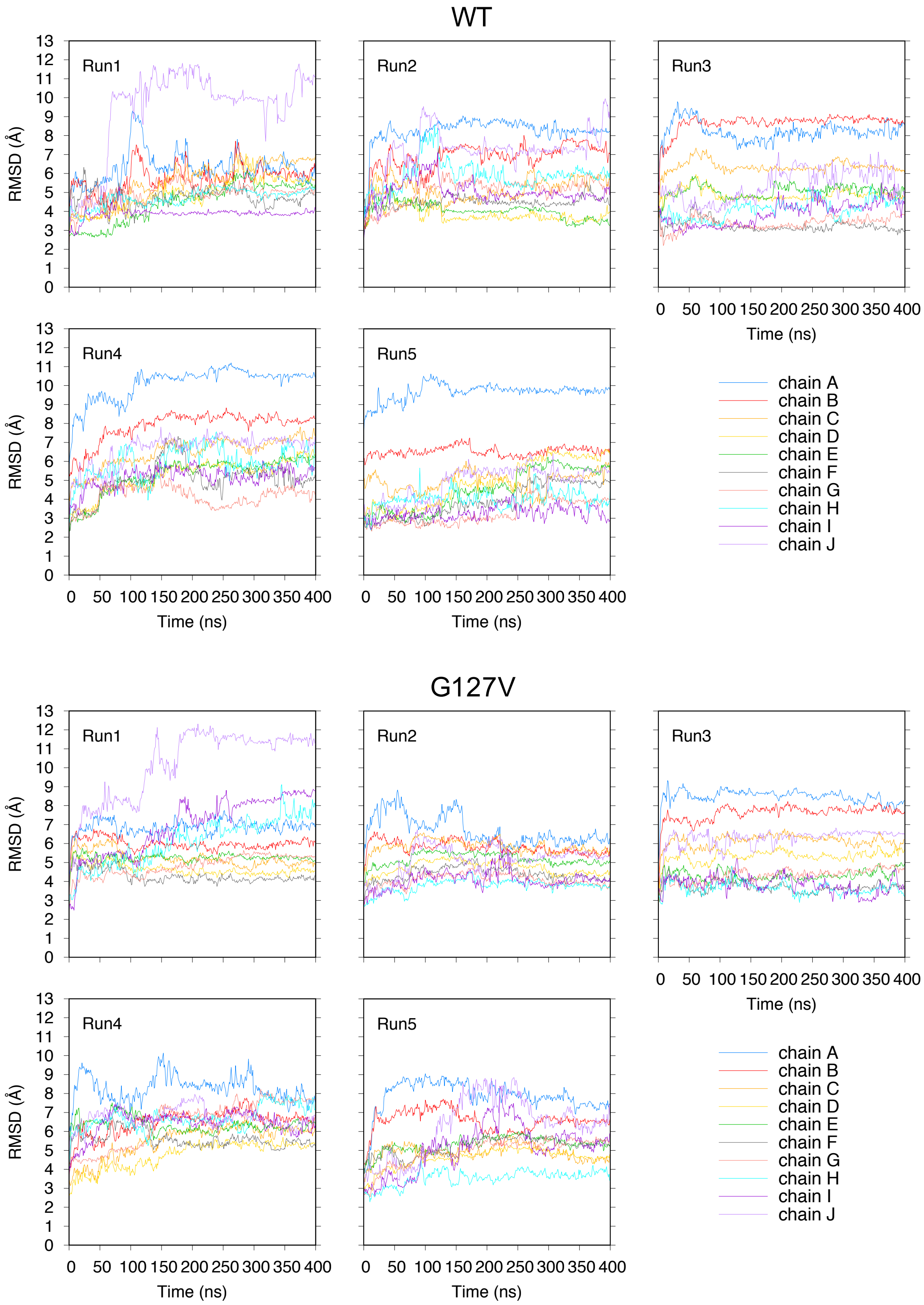

Figure S10: Backbone RMSD of $PrP_{107\text{-}143}$ protofibrils with respect to the constructed model (see Figure 4A).

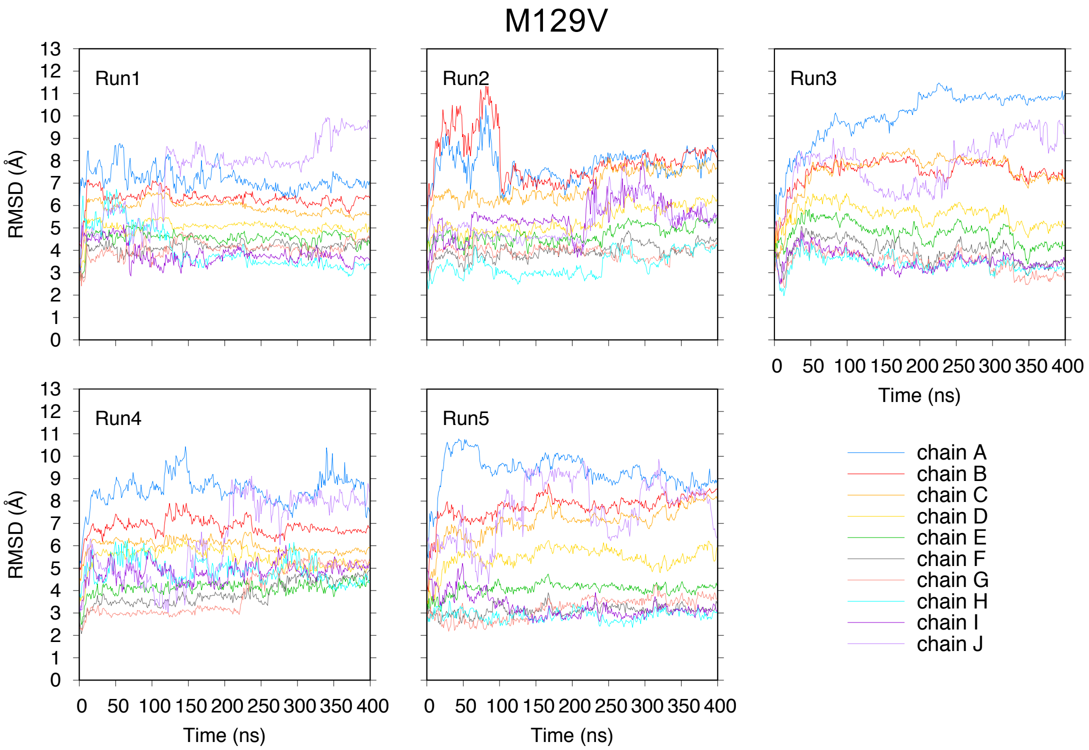

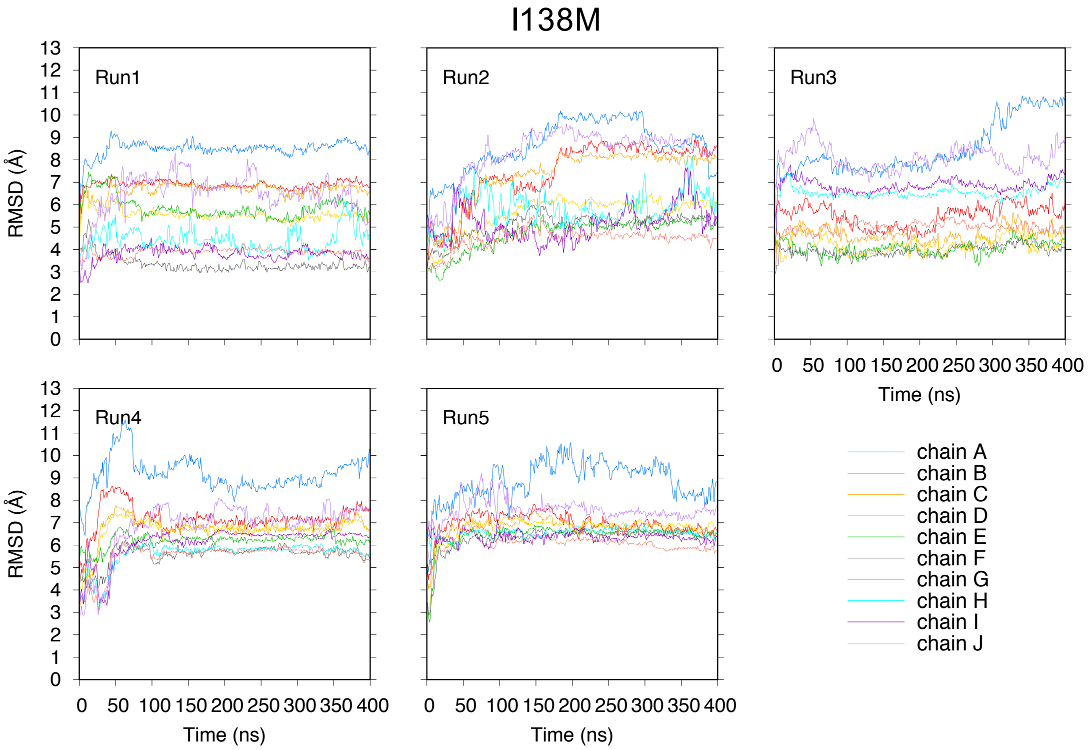

Figure S10: (cont'd.)

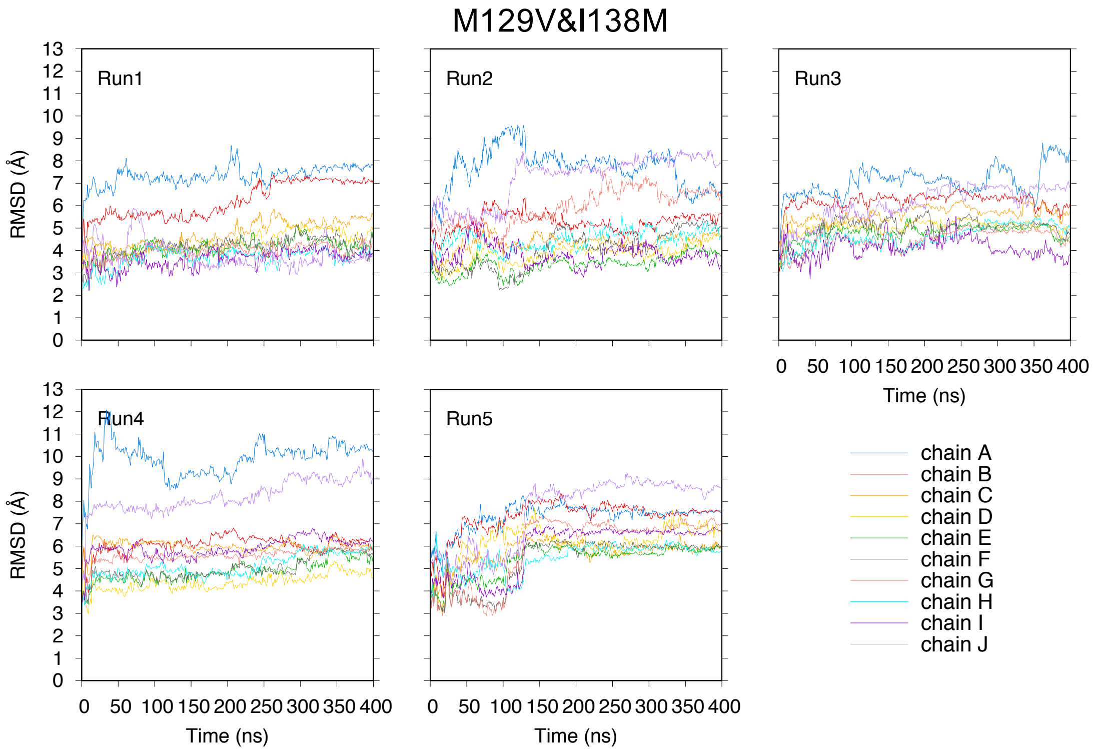

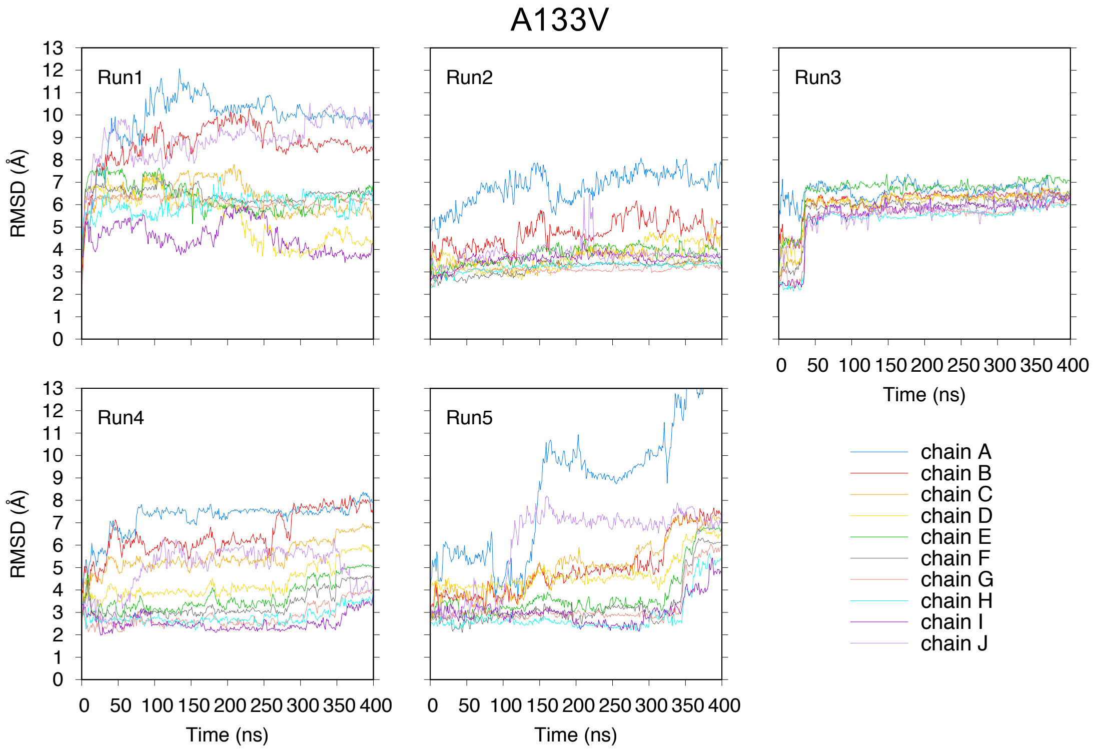

Figure S10: (cont'd.)

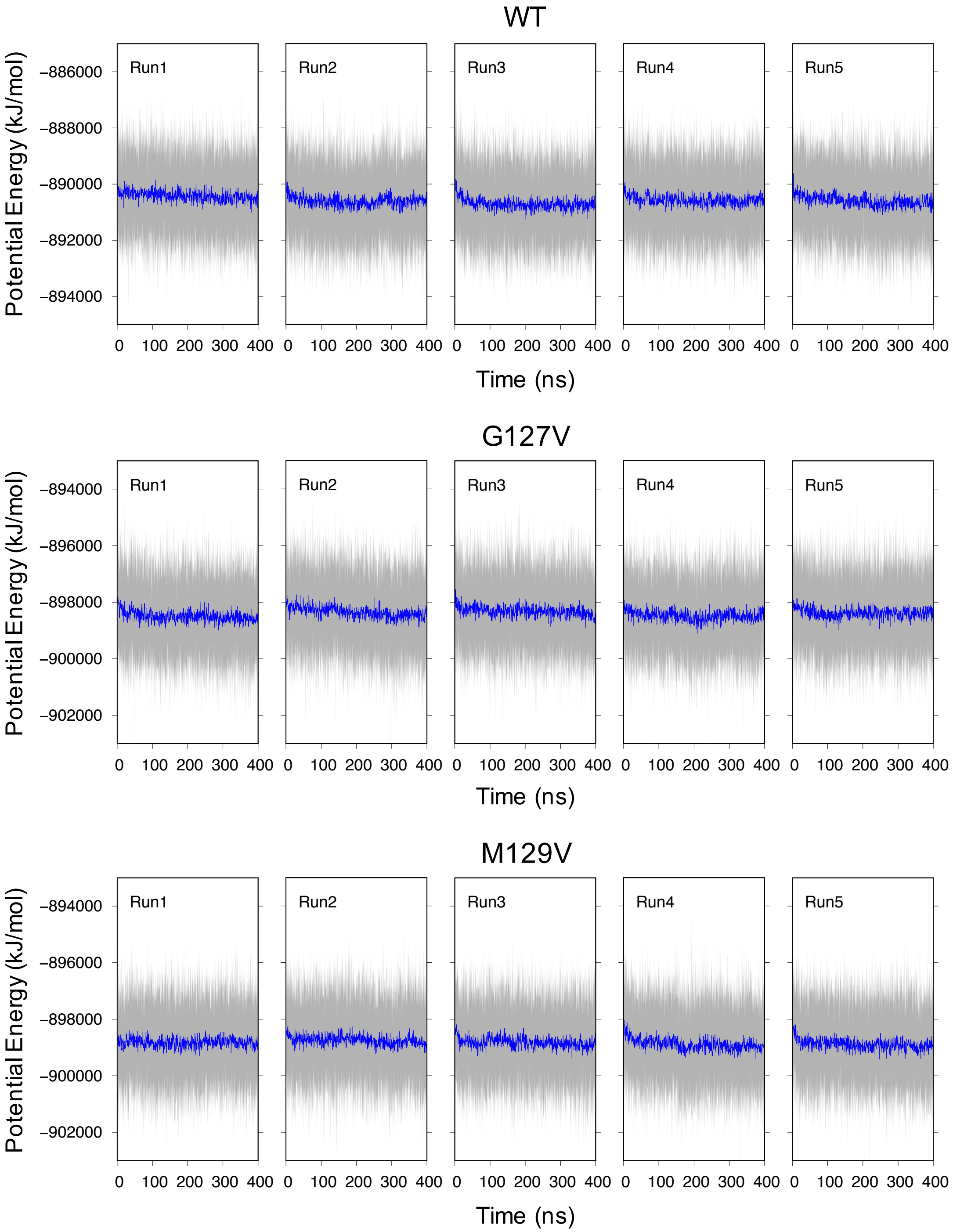

Figure S11: Potential energy as a function of time for $PrP_{107\text{-}143}$ protofibrils (gray). The blue line represents a running average over 500 ps.

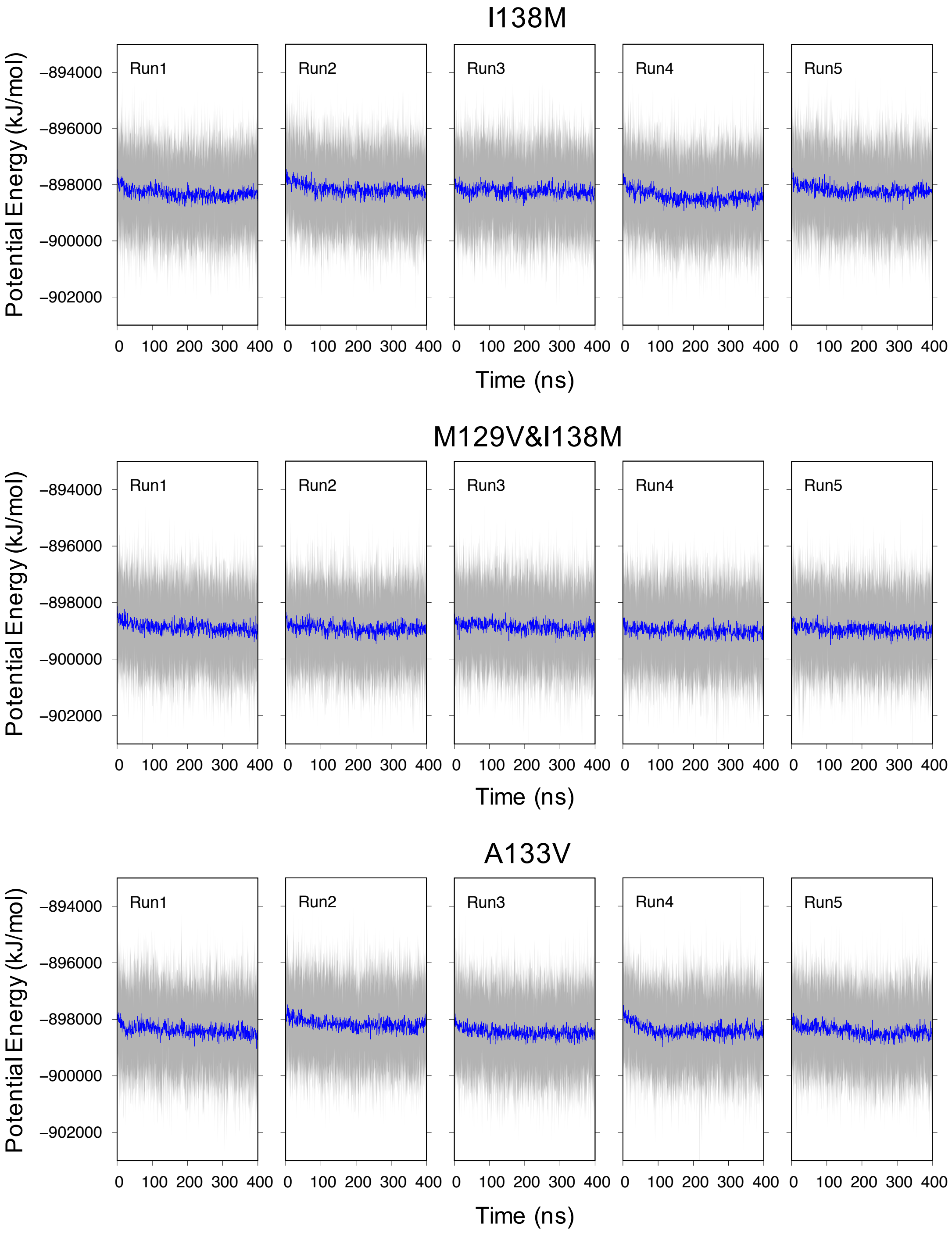

Figure S11: (cont'd.)

References

(1) Salamanca Viloria, J.; Allega, M. F.; Lambrughi, M.; Papaleo, E. An optimal distance cutoff for contact-based Protein Structure Networks using side-chain centers of mass. *Sci. Rep.* **2017**, *7*, 2838.

(2) Tuttle, M. D.; Comellas, G.; Nieuwkoop, A. J.; Covell, D. J.; Berthold, D. A.; Kloepper, K. D.; Courtney, J. M.; Kim, J. K.; Barclay, A. M.; Kendall, A. et al. Solid-state NMR structure of a pathogenic fibril of full-length human α-synuclein. *Nat. Struct. Mol. Biol.* **2016**, *23*, 409–415.
